# Prenatal whole-exome sequencing for fetal structural anomalies: a retrospective analysis of 145 Chinese cases

**DOI:** 10.1186/s12920-023-01697-3

**Published:** 2023-10-25

**Authors:** Yayun Qin, Yanyi Yao, Nian Liu, Bo Wang, Lijun Liu, Hui Li, Tangxinzi Gao, Runhong Xu, Xiaoyan Wang, Fanglian Zhang, Jieping Song

**Affiliations:** 1 Medical Genetics Center, Maternal and Child Health Hospital of Hubei Province, Wuhan, 430070 Hubei Province China; 2 Honghu Hospital of Traditional Chinese Medicine, Jingzhou, 433200 Hubei Province China

**Keywords:** Whole-exome sequencing, Prenatal diagnosis, Structural anomalies, Prenatal ultrasonography

## Abstract

**Background:**

Whole-exome sequencing (WES) significantly improves the diagnosis of the etiology of fetal structural anomalies. This study aims to evaluate the diagnostic value of prenatal WES and to investigate the pathogenic variants in structurally abnormal fetuses.

**Methods:**

We recruited 144 fetuses with structural anomalies between 14 and 2020 and 15 December 2021 in the study. Genetic screening was performed by WES combined with karyotyping and chromosomal microarray analysis. The molecular diagnostic yield of prenatal WES for each type of fetal structural anomaly and the identified pathogenic genes and mutations were reported.

**Results:**

In this study, we retrospectively analyzed the clinical and genetic data of 145 structurally anomalous fetuses. These cases were classified into 9 phenotypic classes based on antenatal ultrasound findings. Thirty-eight pathogenic variants in 24 genes were identified in 35 of the 145 cases, including 14 novel variants in 13 genes (*EP300*, *MYH3*, *TSC2*, *MMP9*, *CPLANE1*, *INVS*, *COL1A1*, *EYA1*, *TTC21B*, *MKS1*, *COL11A2*, *PDHA1* and *L1CAM*). Five additional pathogenic variants were classified as incidental findings. Our study showed that the overall diagnosis rate of WES was 28.1% (27/96) in the parent-fetus trio cases and 16.3% (8/49) in the proband-only cases. Fetuses with musculoskeletal anomalies had the highest diagnostic yield (51.4%, 19/37). In addition, *FGFR3* and *COL1A1* were the most common pathogenic genes.

**Conclusions:**

Our work expands the mutation spectrum of the genes associated with fetal structural anomalies and provides valuable information for future parental genetic counselling and pregnancy management of the structurally anomalous fetuses.

**Supplementary Information:**

The online version contains supplementary material available at 10.1186/s12920-023-01697-3.

## Background

Prenatal ultrasound is a routine screening method for fetal malformations, identifying fetal structural anomalies in approximately 3% of fetuses [1]. The anomalies seen in different tissues and organs of the body are commonly associated with a variety of diseases, including heart defects, skeletal dysplasia, congenital anomalies of the kidneys and urinary tracts (CAKUT), craniofacial anomalies, and nervous system abnormalities. However, due to the limitations of prenatal ultrasonography, it is often difficult to fully determine the fetal phenotype, making the diagnosis of fetal structural anomalies more challenging.

Fetal structural anomalies are usually caused by pathogenic sequence variants and chromosomal aberrations (such as chromosomal aneuploidies and copy number variants) in development-related genes [2]. Soft ultrasound markers, such as increased nuchal translucency, thickened nuchal skin fold, amniotic fluid abnormalities, and single umbilical artery, may also indicate a risk for genetic disease [3]. In prenatal genetic testing, sequence variants are often detected by whole-exome sequencing (WES), while chromosomal aberrations are detected by karyotyping and chromosomal microarray analysis (CMA). Accurate genetic diagnosis is essential not only for the obstetricians to assess the prognosis of fetuses with ultrasound anomalies, but also for the parents to make an informed decisions about pregnancy. It is also helpful in the planning of neonatal management. However, due to the high degree of clinical and genetic heterogeneity and the fact that the vast majority of cases are sporadic, it is extremely challenging to determine the disease-causing variants responsible for the fetal structural anomalies.

WES has become a powerful and cost-effective diagnostic approach for genetic disorders [4, 5]. Several studies have reported the successful application of WES in the prenatal genetic diagnosis of fetal structural anomalies in combination with karyotyping and CMA [6–14]. The diagnostic yield of prenatal WES ranged from 8.5 to 39.4% in different studies [15–19]. Many disease-causing genes, such as *LBR*, *SLC26A2*, *FLNB*, *COL1A2*, *COL2A1*, and *FGFR3*, have been found by WES to be frequently associated with skeletal dysplasia in fetuses. Nevertheless, incomplete phenotypic identification due to the limitations of ultrasound examination and insufficient data on the functions of many candidate genes make it very difficult to determine the clinical significance of variants identified by WES [20–22]. The underlying genetic causes of more than 60% of the cases with fetal structural anomalies are still unknown. More phenotypic and genotypic data will improve the accuracy of genetic interpretation and increase our understanding of the pathogenesis of fetal structural anomalies.

In this study, we retrospectively analyzed the clinical and genetic data of 145 structurally anomalous fetuses. These cases were classified into 9 phenotypic classes based on antenatal ultrasound findings. Genetic screening was performed by WES combined with karyotyping and CMA. The molecular diagnostic yield of prenatal WES for each type of fetal structural anomaly and the identified pathogenic genes and mutations were reported. These data provide valuable information for advancing the clinical application of prenatal WES and for establishing systemic genotype-phenotype associations in prenatal diagnosis.

## Methods

### Participants recruitment and DNA preparation

From 14 to 2020 to 15 December 2021, we recruited 145 pregnancies in which the fetuses showed structural anomalies during 11–32 weeks by prenatal ultrasonography at the medical genetics centre of the Maternal and Child Health Hospital (Hubei, China). The fetuses had all undergone common tests such as intrauterine infection and autoimmune screening at the hospital by obstetricians. Only those fetuses with no clear cause underwent further genetic screening. Clinical information including maternal age, gestational age, family history, and obstetric history were recorded in detail. This study was approved by the institutional ethics committee. All parents of the fetuses agreed to participate in the study and provided signed informed consent. Clinical geneticists were mainly responsible for interviewing participants, collecting data/samples and providing pre- and post-test genetic counselling to participants. Human phenotype ontology (HPO) terms were used to standardize clinical features in the study.

Sample extraction was performed at different stages depending on the situation. For most pregnant women, amniocentesis was performed at 18–32 weeks of gestation to collect amniotic fluid. A few pregnant women underwent chorionic villus sampling at 12–14 weeks of gestation. Genomic DNA was extracted from amniotic fluid/ chorionic villi, and peripheral blood samples from both biological parents using the Qiagen DNA Blood Midi/ Mini Kit (Qiagen GmbH, Hilden, Germany) according to the manufacturer’s protocol.

### Whole-exome sequencing and bioinformatics analysis

WES of parent-fetus trios (fetuses and parents) and proband-only (fetuses) was performed on DNA samples at Berry Genomics Corporation. WES samples are also subjected to short tandem repeats (STR) to exclude maternal contamination for parentage analysis. Massively parallel sequencing was performed using NanoWES (Berry Genomics, China) and the Novaseq6000 platform (Illumina, San Diego, USA). The following metrics were achieved for all samples: mean coverage depth was ~ 100×, and ~ 95% of target bases (exons and ± 20 intronic nucleotides flanking the exon-intron boundaries of all nuclear genes) were interrogated at > 20× read depth.

Raw image files were processed using CASAVA v1.82 for base calling to generate raw data. The raw sequence data were aligned to the human reference genome (hg38) using the Burrows-Wheeler Aligner tool. The PCR duplicates were removed using Picard v1.57 (http://picard.sourceforge.net/). Single nucleotide variants (SNV) and insertion/deletion (InDel) variants were detected using the Verita Trekker® Variants Detection System and GATK (https://software.broadinstitute.org/gatk/). Detected variants were annotated using ANNOVAR (http://annovar.openbioinformatics.org/en/latest/) and Enliven® Variants Annotation Interpretation System, authorized by Berry Genomics. Nonsynonymous, splicing, and InDel variants were filtered by the 1000 Genome Project (http://browser.1000genomes.org), gnomAD (http://gnomad.broadinstitute.org/), Berrybig data population database, dbSNP (http://www.ncbi.nlm.nih.gov/snp) etc. Variants with a minor allele frequency (MAF) > 0.01 were discarded. The whole exome was analyzed and late onset disorders were filtered out for each case in this study.

### Variant interpretation and reporting

Relevant inheritance patterns, family history and clinical manifestations were considered during variant interpretation. Variants relevant to the corresponding phenotypes were evaluated for pathogenicity according to the adapted American College of Medical Genetics and Genomics (ACMG) guidelines (Richards et al., 2015). Genetic variants were screened under the combined consideration of pathogenicity prediction using multiple tools (REVEL, SIFT, Polyphen2, FATHMM, CADD, Mutation Assessor, SPIDEX, etc.), relative hits from different databases (OMIM, http://www.omim.org; HGMD, http://www.hgmd.org; ClinVar, http://www.ncbi.nlm.nih.gov/clinvar; and HPO, https://hpo.jax.org/app/) and scientific reports. Sanger sequencing was used to confirm the inheritance of the likely pathogenic/ pathogenic variants for parent-fetus trios and proband-only cases. CNV-calling was performed using Sprinkle, a comprehensive tool developed by Berry Genetics. More specifically, XHMM PCA was used to remove sequencing background. CNVKit fixed module was used for GC and bias correction, copy number calculation in exons and long fragment regions, and CNV identification. The window size was 200 bp and the normal reference included 30 samples. Interpretation was based on CNV type, size, frequency, number of key genes and recurrence region according to ACMG guidelines. DECIPHER (https://www.deciphergenomics.org/), DGV (http://dgv.tcag.ca/dgv/app/home) and ClinGen (https://dosage.clinicalgenome.org/) were used to assess CNV pathogenicity. Copy number variants (CNVs) identified by WES were further investigated by quantitative PCR.

To report the genetic diagnostic result for each fetus, clinical phenotype-related and genetic pattern compatible variants with sufficient evidence of pathogenicity were included in the main report. Variants related to the prenatal indications but with insufficient evidence of inheritance pattern and/or pathogenicity were included in the secondary report. Variants of uncertain significance (VUS) were occasionally included when there was a strong indication for reporting (e.g., the VUS was compound heterozygous with a pathogenic variant). The highly penetrant pathogenic variants that were unrelated to the fetal phenotypes were classified as incidental findings. These variants were predicted to cause moderate to severe childhood onset disorders, such as neurodevelopmental disorders and metabolic disorders. Additional relevant tests were usually carried out on the fetuses before or after birth. The results are explained in detail to the parents by clinical geneticists. Statistical comparisons were made using the Chi-square test.

## Results

### Sample characteristics

The 145 cases were classified into 9 phenotypic groups based on the ultrasound results (Table S1). Most cases showed abnormalities in the musculoskeletal system (*n* = 37), multisystem (*n* = 36), genitourinary system (*n* = 24), and nervous system (*n* = 13) (Table 1). Other common soft ultrasound markers including increased nuchal translucency, thickened nuchal skin fold, amniotic fluid abnormalities, and single umbilical artery, were categorized into miscellaneous abnormalities of the prenatal birth and development group (*n* = 12). The 145 cases include 96 fetus-parental trios and 49 proband-only cases. In all cases, the chromosomal aberrations were excluded by means of karyotyping and CMA.

**Table 1:** Proportion of diagnostic genetic variants identified in fetuses with each phenotypic abnormality

Phenotype Category	Trio cases	Positive trio cases	Positive rate of trio cases	Proband-only cases	Positive proband-only cases	Positive rate of proband-only cases
Musculoskeletal system	25	14	56%	12	5	41.7%
Abnormalities of multisystem	21	8	38.1%	15	0	
Genitourinary system	18	2	11.1%	6	1	16.7%
Nervous system	10	1	10%	3	1	33.3%
Other abnormalities of prenatal birth	8	1	12.5%	4	1	25%
Craniofacial abnormalities	7	1	14.3%	4	0	
Cardiovascular system	5	0		3	0	
Abnormality of the abdomen	1	0		1	0	
Digestive system	1	0		1	0	
Total	96	27	28.1%	49	8	16.3%

### WES molecular diagnostic rate

There were 35 cases identified by WES with likely pathogenic/pathogenic variants closely associated with the abnormal fetal phenotypes, resulting in an overall diagnostic rate of 24.1% (35/145). Four cases (2.8%, 4/145) carrying 5 likely pathogenic/pathogenic variants that were not considered phenotypically correlated were classified as incidental findings. In the parent-fetus trio cases (*n* = 96), the diagnostic rate of WES was 28.1% (27/96). In contrast, the diagnostic rate of WES in the proband-only cases (*n* = 49) was 16.3% (8/49) (Table 1 and and Supplementary file 1). The diagnostic rate of the parent-fetus trio group was significantly higher than that of the proband-only group (*p* < 0.05). In addition, variants were classified as VUSs in 13 cases (Table S2). The proportion of VUSs in the proband-only samples (14.3%, 7/49) was higher than that of the fetus-parental trios (6.25%, 6/96). The consequences of these variants have not yet been determined.

**Fig. 1:**
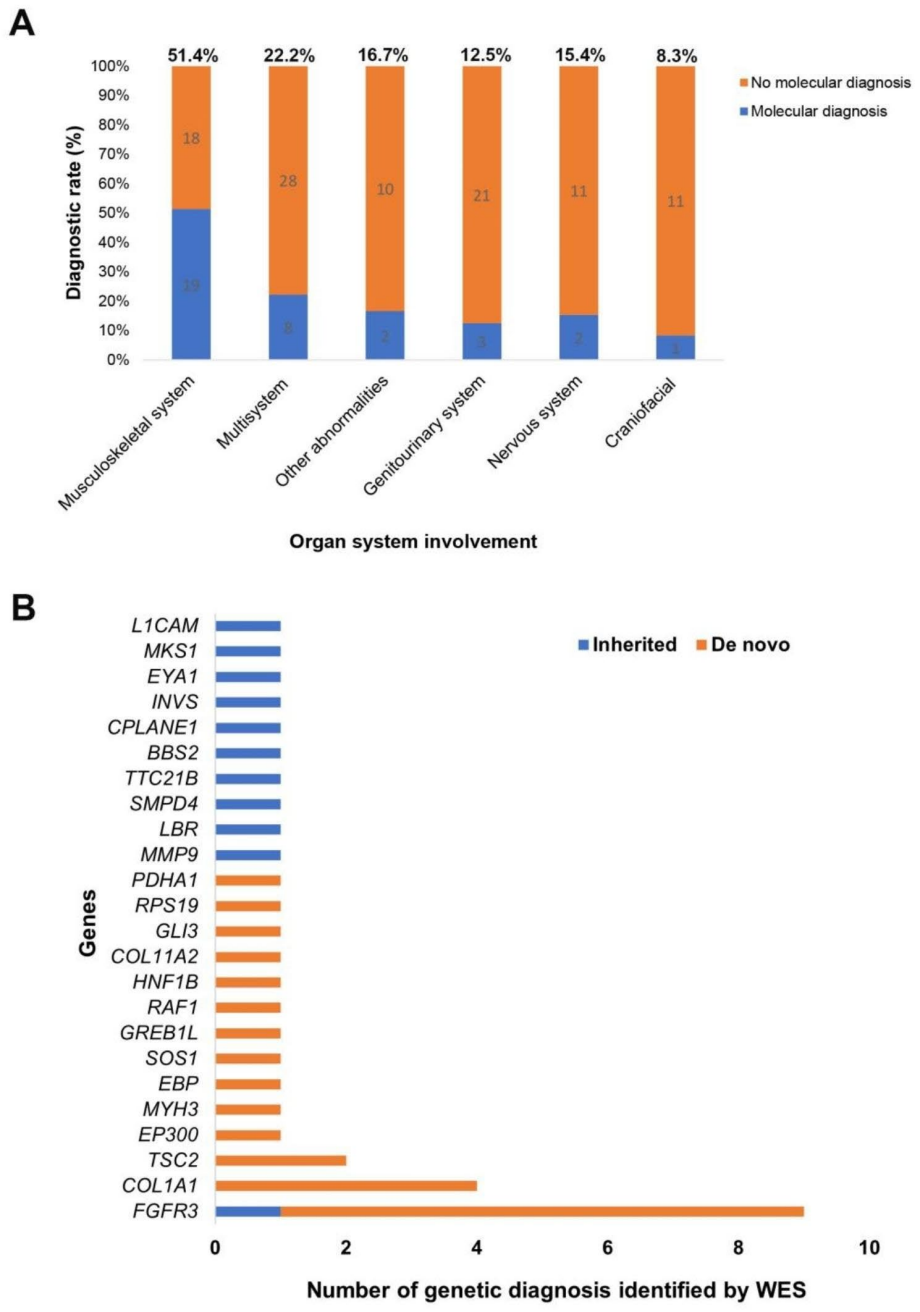
Characteristics of the potential diagnoses in fetuses with structural abnormalities. **A** Proportion of diagnostic genetic variants identified in fetuses with each phenotypic abnormality. **B** Potential diagnostic gene and gene frequency detected by WES

The diagnostic yield varied between the phenotypic groups (Fig. 1A), with the highest diagnostic yield in the musculoskeletal system anomalies (51.4%, 19/37), followed by multisystem anomalies (22.2%, 8/36), other prenatal anomalies (16.7%, 2/12), genitourinary system anomalies (12.5%, 3/24) and nervous system anomalies (15.4%, 2/13). A low yield of genetic diagnosis was observed in the group of craniofacial anomalies (8.3%, 1/12).

### Mutation type and inheritance pattern of the pathogenic variants

A total of 38 different pathogenic variants in 24 genes were identified in 35 of the 145 cases, including 8 frameshifts, 2 stopgains, 5 splicings, 2 in-frame deletions, 19 nonsynonymous changes, 1 start_lost and 1 CNV (Table 2). Compound heterozygous variants were detected in 7 cases (Table 2). Of the 35 cases, 68.6% (*n* = 24) were autosomal dominant (AD), 22.8% (*n* = 8) were autosomal recessive (AR), and 8.6% (*n* = 3) were X-linked (XL) (Table 3). Most AD cases were associated with *de novo* variants (75%, *n* = 18/24; Table 3), whereas most AR cases were associated with compound heterozygous variants (87.5%, *n* = 7/8; Table 3).

**Table 2:** Summary of likely pathogenic/ pathogenic variants identified by WES in fetuses with structural anomalies

Case ID	Sonographic findings	Phenotype category	Gene	Variant	Inheritance	Zygosity	Clinical significance	Disease
**Fetus-parental trios**								
3084	Short long bone	Musculoskeletal system	*FGFR3*	NM_000142.4: c.1138G > A p.(G380R)	AD	hom (affected parents)	P	Achondroplasia MIM:100,800
3033	Short nasal bone, single umbilical artery, nasal skin thickening, abnormal morphology of bilateral auricles	Craniofacial abnormalities	*EP300*	NM_001429.4: c.5527dup▲ p.(Q1843Pfs*40)	AD	de novo het	P	Menke-Hennekam syndrome 2 MIM:618,333; Rubinstein Taybi syndrome 2 MIM:613,684
2964	Skeletal dysplasia, short long bones	Musculoskeletal system	*LBR*	NM_002296.4: c.1757G > A p.(R586H) c.1748G > A p.(R583Q)	AR	compound het	LP P	Greenberg skeletal dysplasia MIM:215,140
2949	Cystic hygroma, lymphocysts, hydrops fetalis, pleural effusion, flexion contracture, clubfoot	Multiple system	*MYH3*	NM_002470.4: c.3247_3248 + 1del▲	AD	de novo het	LP	Arthrogryposis, distal, type 2 A MIM:193,700
5540	Lateral ventricle dilatation, ventricular abnormality, cardiac rhabdomyoma	Multiple system	*TSC2*	NM_000548.5: c.5239_5256del▲ p.(I1747_Q1752del)	AD	de novo het	P	Tuberous sclerosis-2 MIM:613,254
2997	Renal dysplasia, cranial abnormalities	Genitourinary system	*BBS2*	NM_031885.4: c.700 C > T p.(R234*)	AR	hom	P	Bardet-Biedl syndrome 2 MIM:615,981
8222	Short long bone	Musculoskeletal system	*EBP*	NM_006579.3: c.278 A > T p.(D93V)	XLD	de novo het	LP	Chondrodysplasia punctata, X-linked dominant MIM:302,960
2090	Hypoplasia of the lower limbs	Musculoskeletal system	*MMP9*	NM_004994.3: c.151 C > T▲ p.(R51C) c.929del▲ p.(G310Afs*28)	AR	compound het	LP LP	Metaphyseal anadysplasia 2 MIM:613,073
2079	Short limb deformity, short long bone, bowed femur, polyhydramnios	Musculoskeletal system	*FGFR3*	NM_000142.4: c.1138G > A p.(G380R)	AD	de novo het	P	Achondroplasia MIM:100,800
6431	Widen cerebellar medullary pool, left renal cyst, bipedal polydactyly	Multiple system	*CPLANE1*	NM_023073.3: c.2854_2855insCT▲ p.(N952Tfs*13) c.3599 C > T p.(A1200V)	AR	compound het	LP LP	Joubert syndrome 17 MIM:614,615 Orofaciodigital syndrome VI MIM:277,170
8232	Widen cerebellar medullary pool, abnormal renal morphology, oligohydramnios	Multiple system	*INVS*	NM_014425.5: c.796 + 5G > A c.805_806del▲ p.(Q269Dfs*10)	AR	compound het	P P	Nephronophthisis 2, infantile MIM:602,088
2051	Short long bone, abnormality of the thorax	Musculoskeletal system	*COL1A1*	NM_000088.3: c.2110G > A p.(G704S)	AD	de novo het	P	Osteogenesis imperfecta MIM:166,210
2014	Arthrogryposis	Musculoskeletal system	*SMPD4*	NM_017951.4: c.387-1G > A wes[hg38]chr2(13 0142742-130202459)x1 59.72 kb deletion	AR	compound het	P LP	Neurodevelopmental disorder with microcephaly, arthrogryposis, and structural brain anomalies MIM:618,622
6411	Short femur length	Musculoskeletal system	*FGFR3*	NM_000142.4: c.1620 C > A p.(N540K)	AD	de novo het	P	Achondroplaia MIM:100,800
6429	Femoral fracture	Musculoskeletal system	*COL1A1*	NM_000088.3: c.3977T > G▲ p.(F1326C)	AD	de novo het	LP	Osteogenesis imperfecta MIM:166,210
8233	Hypoplastic auricles, Webster’s cavity, polyhydramnios	Multiple system	*EYA1*	NM_000503.6: c.1698 + 2T > C▲	AD	het (affected father)	LP	Otofaciocervical syndrome MIM:166,780
5549	Short long bone	Musculoskeletal system	*TTC21B*	NM_024753.5: c.3130_3131dup▲ p.(H1045Dfs*20) c.2569G > A p.(A857T)	AR	compound het	LP LP	Short-rib thoracic dysplasia 4 with or without polydactyly MIM:613,819
5509	Developmental malformation	Multiple system	*MKS1*	NM_017777.4: c.1407 + 66G > A▲ c.1411dup p.(E471Gfs*178)	AR	compound het	VUS LP	Meckel syndrome 1 MIM:249,000
8461	Increased echo in parenchyma of both kidneys, left hydronephrosis with extrarenal pelvis dilatation, polyhydramnios	Genitourinary system	*HNF1B*	NM_000458.4: c.884G > A p.(R295H)	AD	het (affected father)	LP	Renal cysts and diabetes Syndrome MIM:137,920
9445	Short limbs	Musculoskeletal system	*COL11A2*	NM_080680.3: c.3850 C > T▲ p.(R1284W)	AD	de novo het	LP	Fibrochondrogenesis 2 MIM:614,524
1768	Curved femur, osteogenesis hypoplasia	Musculoskeletal system	*COL1A1*	NM_000088.4: c.1084G > A p.(G362S)	AD	het (affected father)	LP	Osteogenesis imperfecta MIM:166,200
1781	Polydactyly in the left hand, polydactyly in both feet toes	Musculoskeletal system	*GLI3*	NM_000168.6: c.868 C > T p.(R290*)	AD	de novo het	P	Greig cephalopolysyndactyly syndrome MIM:175,700
0602	Increased nuchal translucency (5.9 mm), intrauterine growth retardation	Other abnormalities of prenatal birth	*RPS19*	NM_001022.4: c.3G > T p.(M1?)	AD	de novo het	P	Diamond-Blackfan anemia 1 MIM:105,650
0646	Widen lateral ventricles, anterior horn hypoechoic, widen pellucid septum, hypoplasia of corpus callosum	Nervous system	*PDHA1*	NM_000284.4: c.766G > A▲ p.(G256R)	XLD	de novo het	LP	Pyruvate dehydrogenase E1-alpha deficiency MIM:312,170
5588	Short femur, Short humerus, polyhydramnios	Musculoskeletal system	*FGFR3*	NM_000142.4: c.1138G > A p.(G380R)	AD	de novo het	P	Achondroplasia MIM:100,800
5539	The left posterior frontal gyri was deepened and thicken the cerebral cortex	Multiple system	*TSC2*	NM_000548.5: c.5238_5255del p.(H1746_R1751 del)	AD	de novo het	P	Tuberous sclerosis-2 MIM:613,254
0543	Curved femur, short femur	Musculoskeletal system	*COL1A1*	NM_000088.4: c.1678G > A p.(G560S)	AD	de novo het	P	Osteogenesis imperfecta MIM:166,200
**Proband- only samples**								
3078	Short long bone	Musculoskeletal system	*FGFR3*	NM_000142.4: c.1138G > A p.(G380R)	AD	de novo het	P	Achondroplasia MIM:100,800
3031	Lethal short limb deformity, short limbs, small chest	Musculoskeletal system	*FGFR3*	NM_000142.4: c.1108G > T p.(G370C)	AD	de novo het	P	Thanatophoric dysplasia MIM:187,600
3005	Hydrocephalus	Nervous system	*L1CAM*	NM_001278116.2: c.2555del▲ p.(Y852Sfs*129)	XLR	hemi (unaffected mother)	LP	Hydrocephalus due to aqueductal stenosis MIM:307,000
2919	Lethal short limb deformity, short fingers, small chest, lung dysplasia	Multiple system	*FGFR3*	NM_000142.4: c.1118 A > G p.(Y373C)	AD	de novo het	P	Thanatophoric dysplasia MIM:187,600
4714	Short long bone, duplicate kidney, polyhydramnios	Multiple system	*FGFR3* *SOS1*	NM_000142.4: c.1138G > A p.(G380R) NM_005633.4: c.1654 A > G p.(R552G)	AD AD	de novo het de novo het	P P	Achondroplasia MIM:100,800 Noonan syndrome 4 MIM:610,733
8491	Left renal agenesis	Genitourinary system	*GREB1L*	NM_001142966.3: c.4881_4882del p.(H1627Qfs*17)	AD	de novo het	LP	Renal hypodysplasia/aplasia 3 MIM:617,805
1752	Increased nuchal translucency (6.8 mm), cystic hygroma, polyhydramnios	Other abnormalities of prenatal birth	*RAF1*	NM_002880.4: c.770 C > T p.(S257L)	AD	de novo het	P	Noonan syndrome 5 MIM:611,553
4743	Short femur, Short humerus, polyhydramnios	Musculoskeletal system	*FGFR3*	NM_000142.4: c.1138G > A p.(G380R)	AD	de novo het	P	Achondroplasia MIM:100,800

Abbreviations: AD, autosomal dominant; AR, autosomal recessive; Hemi, hemizygous; Het, heterozygous; Hom, homozygous; LP, likely pathogenic; P, pathogenic; XLD, X-linked dominant; XLR, X-linked recessive. ▲, novel identified variants

**Table 3:** Inheritance patterns of genes that identified by molecular diagnostics

	All cases (*n* = 145)	Trios (*n* = 96)	Proband-only (*n* = 49)
Autosomal dominant (AD)	24	17	7
De novo	18	14	7
Inherited	6	3	0
Autosomal recessive (AR)	8	8	0
Compound heterozygous	7	7	0
Homozygous	1	1	0
X-linked dominant (XLD)	2	2	0
X-linked recessive (XLR)	1	0	1
Total molecular diagnoses	35	27	8

### Novel variants identified in the definitive diagnostic cases

Importantly, 14 novel pathogenic variants were identified in the study (Table 2). Five novel pathogenic/likely pathogenic variants, including the c.151 C > T and c.929del in *MMP9*, c.3977T > G in *COL1A1*, c.3130_3131dup in *TTC21B*, and c.3850 C > T in *COL11A2* were found in 4 cases with skeletal dysplasia. Six novel pathogenic/likely pathogenic variants, including c.3247_3248 + 1del in *MYH3*, c.5239_5256del in *TSC2*, c.2854_2855insCT in *CPLANE1*, c.805_806del in *INVS*, c.1698 + 2T > C in *EYA1* and c.1407 + 66G > A in *MKS1*, were found in six cases with multiple abnormalities. Two novel variants in *L1CAM* (c.3130_3131dup) and *PDHA1* (c.766G > A) were detected in two cases with central nervous system abnormalities. A pathogenic variant, c.5527dup in *EP300*, was identified in the case with craniofacial abnormalities.

### Representative diagnostic cases using prenatal WES

A pathogenic splicing variant in *SMPD4* in trans with a deletion of 59.72 kb (chr2: 130,142,742–130,202,459) spanning *SMPD4* was found in a fetus with arthrogryposis (case 2014, Table 2) using WES-based CNV analysis. *SMPD4* is the gene responsible for a developmental disorder characterized by microcephaly and congenital arthrogryposis [23], which was consistently observed in the aforementioned case.

#### Case 2997

carried a homozygous stopgain variant in *BBS2* inherited from his parents. This mutation has been previously reported in patients affected with Bardet-Biedl syndrome 2. In addition to renal dysplasia, *BBS2* mutations often cause retinitis pigmentosa, a progressive retinal degenerative disease that cannot be observed in the prenatal period. According to the family history, the proband’s brother has mild intellectual disability and night blindness (as early symptoms of retinitis pigmentosa). The clinical significance of the *BBS2* variant was further determined by the family history.

Pathogenic variants in *FGFR3* are known to cause autosomal dominant achondroplasia and hypochondroplasia. However, a homozygous missense variant in *FGFR3* was identified in one case (case 3084) with shortened long bones (Table 2). Further pedigree analysis revealed that the homozygous *FGFR3* variant (c.1138G > A, p.G380R) was inherited from the proband’s parents affected by dwarfism. Two pathogenic gene variants (*FGFR3*, *SOS1*) were detected by WES in a fetus (case 4714) with shortened long bones, multiple kidneys and polyhydramnios by WES. These two diagnoses could clearly explain the structural abnormalities of the fetus.

### Incidental findings

Incidental genetic variations were detected in four cases (Table 4). These variants were predicted to cause conditions unrelated or partially related to the fetal phenotypes. For example, in case 2092 with skin oedema, pleural effusion, and talipes equinovarus, two pathogenic compound heterozygous variants in the *TTN* gene were identified. *TTN* is usually associated with cardiomyopathy and muscular dystrophy, which was not relevant to the ultrasound findings in this case. Another example is a *de novo* heterozygous variant (c.1019 C > T, p.T340M) in *FGFR1*, which was identified in case 8212 with abnormalities of the cerebral ventricles and septum pellucidum and the agenesis of the corpus callosum. However, *FGFR1* mutations are often associated with Hartsfield syndrome, which is characterized by holoprosencephaly, ectrodactyly and cleft lip/palate [24].

**Table 4:** Incidental findings

Case ID	Sonographic findings	Phenotype category	Gene	Variant	Inheritance	Zygosity	Clinical significance	Disease
2907	Abnormal foot posture, clubfoot	Musculoskeletal system	*TRIP12*	NM_001284214.2: c.2065 C > T p.(Q689*)	AD	de novo het	P	Mental retardation, autosomal dominant 49 MIM:617,752
6428	Agenesis of corpus callosum	Nervous abnormalities	*ARID1B*	NM_020732.3: c.6393del p.(M2132Wfs*13)	AD	de novo het	P	Coffin-Siris syndrome 1 MIM:135,900
8212	Abnormality of the cerebral ventricles and the septum pellucidum, agenesis of corpus callosum	Nervous system	*FGFR1*	NM_023110.3: c.1019 C > T p.(T340M)	AD	de novo het	LP	Hartsfield syndrome MIM:615,465
2092	skin edema, pleural effusion, and talipes equinovarus	Multiple system	*TTN*	NM_001267550.2:c.38876-2 A > C c.79,096 A > T p.(K26366*)	AR	compound het	P P	Muscular dystrophy, limb-girdle, autosomal recessive 10 MIM:608,807

Abbreviations: AD, autosomal dominant; AR, autosomal recessive; Het, heterozygous; Hom, homozygous; LP, likely pathogenic; P, pathogenic

A *de novo* heterozygous variant in the *TRIP12* gene was detected in the case 2907 with abnormal feet. Pathogenic variants in *TRIP12* are often associated with neurodevelopmental disorders, such as developmental delay and intellectual disability. The clinical significance of *TRIP12* variants should be confirmed by adequate postnatal follow-up. *ARID1B* pathogenic variants are associated with several clinical features, including intellectual disability, developmental delay, severe speech delay, corpus callosum abnormalities, etc. The *ARID1B* variant was classified as an incidental finding in case 6428, because most of the clinical features associated with neurodevelopmental disorders are difficult to observe on prenatal ultrasound.

### Genes that frequently associated with fetal structural abnormalities

Variants in *FGFR3* (*n* = 9) and *COL1A1* (*n* = 4) were the most common diagnostic findings in this study (Fig. 1B). These variants were all found to be responsible for the phenotypes observed in the fetuses. Four pathogenic missense variants in *FGFR3* were found in 9 cases (case 3084, 2079, 6411, 5588, 3078, 3031, 2919, 4714, 4743) with skeletal dysplasia. The variants c.1138G > A in *FGFR3* were detected six times. Four missense variants in *COL1A1* were detected in four different cases (case 2051, 6429, 1768 and 0543, Table 2) with shortened long bones or osteogenesis hypoplasia.

**Fig. 2:**
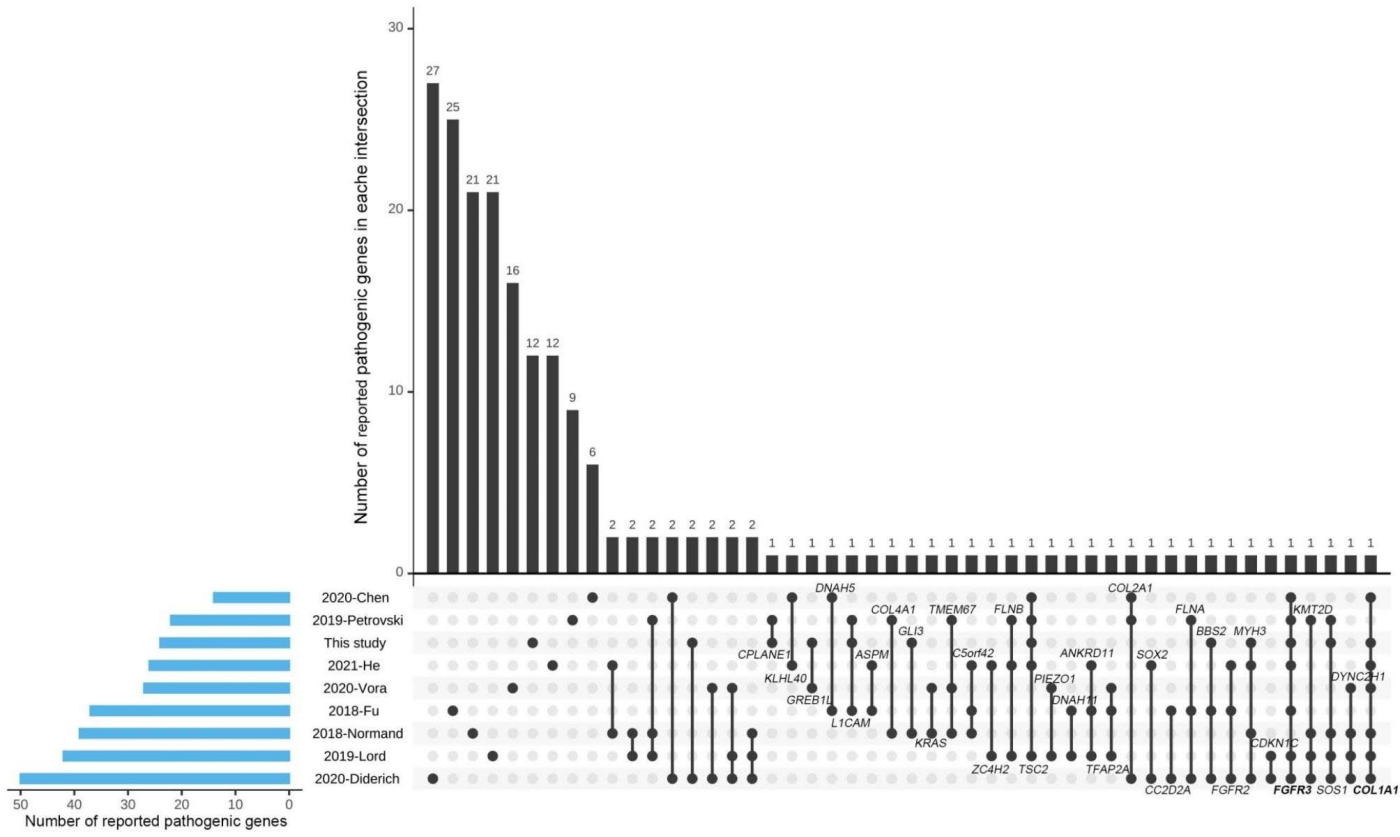
Genes associated with fetal structural abnormalities that have been identified in studies. The horizontal column (blue) represents the number of reported pathogenic genes in each study. The vertical column (black) represents the number of reported pathogenic genes in each intersection. An intersection means a group of pathogenic genes that was shared by one, two, or more studies. The black dots linked by black lines indicate the information that pathogenic genes in each intersection are shared by which studies. The gene symbols in some intersections are labeled

To summarize the pathogenic genes associated with fetal structural abnormalities, we performed a comparative analysis of our results with other 8 recently reported cohort studies that included at least 90 cases of fetal structural abnormalities [13–19, 25]. Among the pathogenic genes identified in our cohort, 12 genes were found to be novel, while 12 genes had already been identified in other studies (Fig. 2). The most frequently identified genes were *FGFR3* and *COL1A1*, which were shared by 7 studies. The genes *TSC2* and *SOS1* were reported in five studies, *DYNC2H1*, *MYH3* and *KMT2D* were detected in four cohort studies. *COL2A1*, *TMEM67*, *BBS2*, *FGFR2*, *L1CAM*, *FLNA*, *FLNB*, *ANKRD11*, *TFAP2A*, *C5orf42*, etc. were found in studies. Details of the disease-causing genes are shown in Fig. 2.

## Discussion

This study reported a retrospective analysis of the clinical and genetic data from 145 structurally anomalous fetuses. A total of 38 pathogenic variants in 24 genes were identified in 35 of the 145 cases, including 14 novel variants in 13 genes. The diagnostic rates for the parent-fetus trios and the proband-only samples were 28.1% (27/96) and 16.3% (8/49), respectively. These findings provide valuable information to promote the clinical application of prenatal WES and to establish genotype-phenotype associations in prenatal diagnosis.

Several recent studies have shown that prenatal WES achieves diagnostic rates of 20–24% in fetuses with structural anomalies with normal karyotype and CMA [16, 26, 27]. In this retrospective study, we found a diagnostic yield of 24.1% (35/145) by WES, which is consistent with the rates reported in studies excluding chromosomal abnormalities. The different diagnostic rates could be affected by many factors, such as cohort size, variant interpretation criteria, diversity of anomalies and inclusion criteria [22]. A molecular diagnosis rate of 28.1% (27/96) was achieved in the fetus-parental trio samples. However, the diagnosis rate was decreased to 16.3% (8/49) in proband-only samples. This indicated that WES with trios would have a higher detection sensitivity than proband-only samples, which is consistent with previous studies [16]. It is likely that trio-WES is more effective in detecting the *de novo* and compound heterozygous variants. Among the 35 cases with a specific molecular diagnosis in this study, the pathogenic variants in 20 cases were *de novo*, accounting for 57.1%. This result suggests that *de novo* variants may be the predominant mutation in fetuses with structural anomalies. In addition, 15 fetuses had pathogenic variants inherited from their parents, suggesting the importance of genetic counselling and carrier screening for pregnant women.

The diagnostic yield of 51.4% gene variants in cases with musculoskeletal anomalies was significantly higher than in other cases with multisystem anomalies (22.2%), nervous system anomalies (15.1%), and genitourinary system anomalies (12.5%). Furthermore, we found a higher diagnostic yield in fetuses with musculoskeletal anomalies (19/37, 51.4%) than in those with an anomaly excluding the skeletal anomalies (8/72, 11.1%). This may be because the fetuses with abnormal skeletal system development have more distinct phenotypic features, just as other researchers have claimed that a specific organ system anomaly may have a higher diagnostic yield than a single structural anomaly [21].

*FGFR3* is a pathogenic gene that causes for achondroplasia with shortened long bones and over 80% of cases have been found with spontaneous gene mutations [28]. Four heterozygous variants in *FGFR3* were identified in 9 cases of skeletal dysplasia. Among these, a homozygous *FGFR3* variant (G380R) in case 3084 was inherited from the parents of the proband affected by dwarfism, which is a rare finding. Six cases carried the same pathogenic variant G380R in *FGFR3.* Of these, two cases carrying the G380R variant (3084, 3078) presented with shortened long bones, and four other *FGFR3* cases (2079, 5588, 4714, 4743) presented with shortened long bones and polyhydramnios. Case 6411, carrying the N540K variant, presented with short femur length only. However, the remaining two cases (3031 and 2919) with thanatophoric dysplasia had short limbs and a small chest. In addition, the *FGFR3* G380R variant was found in the father with dwarfism, but not in the fetus with skeletal dysplasia, indicating the added value of trio WES.

On the other hand, WES can detect incidental findings that cannot be detected by prenatal imaging. For example, neurodevelopmental disorders such as developmental delay and intellectual disability cannot be detected by routine prenatal testing. Case 2907 with abnormal feet was found to carry a *de novo* heterozygous variant in the *TRIP12* gene. Haploinsufficiency of *TRIP12* has been reported to cause childhood-onset neurodevelopmental disorder including intellectual disability [29, 30], as important information cannot be obtained by ultrasound diagnosis alone. The combination of ultrasound imaging with WES-based gene variation detection could avoid the misdetection of fetal anomaly. Nevertheless, without adequate postnatal follow-up, the clinical significance of the *TRIP12* gene variant was uncertain.

Remarkably, a compound heterozygous mutation of pathogenic CNV and SNV in the *SMPD4* gene was identified with the performance of the CNV calling algorithm in WES data analysis (usually for SNV/Indel analysis). This suggested that the WES-based CNV calling tool could be applied as an effective way to improve the diagnostic quality and develop WES as a more powerful method in prenatal diagnosis.

This study reported 14 novel variants with a clinical significance of pathogenic or likely pathogenic among the diagnostic cases. Our study suggests that WES can broaden the mutation spectrum of genes associated with fetal structural anomalies, which would further benefit the prenatal diagnosis and genetic counselling in the future. WES also has the potential to detect new causative genes for specific developmental disorders and to establish new fetal phenotype-genotype correlations in the remaining cases without genetic diagnosis [31].

Comparing our results with eight other recent studies on fetal structural abnormalities showed that *FGFR3* and *COL1A1* are the most common pathogenic genes in musculoskeletal disorders, suggesting a strong genotype-phenotype correlation. Although some pathogenic genes recurred in the test results of different cohorts, the most pathogenic genes are only reported in a single study. The results indicate that the pathogenic molecular mechanism is complicated with a high degree of genetic heterogeneity and that a complete gene network needs to be discovered. WES gene variation should to be further investigated by combining of prenatal and postnatal phenotyping to provide reliable genetic diagnosis [32, 33].

## Conclusions

In this study, WES was used to identify pathogenic gene mutations in fetuses with a wide range of structural anomalies. Our study will not only widen the mutation spectrum of pathogenic gene variations causing fetal structural anomalies, but also provide valuable information for prenatal counselling.

### Electronic supplementary material

Below is the link to the electronic supplementary material.

Supplementary Material 1

Supplementary Material 2

Supplementary Material 3

Supplementary Material 4

## Data Availability

The details of the variant analyzed during the current study are deposited in the ClinVar repository, which is searchable by searching for an SCV accession provided in the summary report. A summary report of our successfully processed data: https://submit.ncbi.nlm.nih.gov/api/2.0/files/cpdivd1g/sub13018827__100__submitter_report_b.txt/?format=attachment. The whole-exome sequencing raw data for the participants are not publicly available due to privacy and ethical restrictions. Other researchers and readers can access these data by requesting from the corresponding author privately.

## Abbreviations

ACMG: American College of Medical Genetics and Genomics
AD: Autosomal dominant
AR: Autosomal recessive
CMA: Chromosomal microarray analysis
CNVs: Copy number variants
HPO: Human phenotype ontology
SNV: Single nucleotide variant
VUS: Variants of uncertain significance
WES: Whole-exome sequencing
XL: X-linked
