# Supplementary material for: Prenatal whole-exome sequencing for fetal structural anomalies: a retrospective analysis of 145 Chinese cases
Source: BMC Med Genomics. 2023 Oct 25;16:262. doi: 10.1186/s12920-023-01697-3 (PMC10601195; doi:10.1186/s12920-023-01697-3)
Supplement: Supplementary file 1 — Supplementary Material 1 [file 12920_2023_1697_MOESM1_ESM.docx]

**Table S1** Phenotype category of fetuses with structural abnormalities

| Phenotype Category | Specific phenotypic abnormalities |
| --- | --- |
| Craniofacial | Cleft lip and palate  Abnormalities of the nose  Abnormalities of the outer ear  Abnormal eye morphology |
| Musculoskeletal system | Shortened long bone  Abnormal vertebral development  Polydactyly  Talipes equinovarus (club foot)  Aplasia/Hypoplasia involving bones of lower limbs  Flexion contracture  Aplasia/Hypoplasia of the extremities  Aplasia/Hypoplasia involving bones of upper limbs  Fatal short limb malformation  Hand clenching  Abnormality of the thorax |
| Miscellaneous abnormalities of prenatal birth and development | Prenatal movement abnormality  Increased nuchal translucency (≥3.5mm with/without cystic hygroma)  Single umbilical artery  Thickened nuchal skin fold  Hydrops fetalis  Abnormality of amniotic fluid  Fetal growth restriction  Abnormalities of placenta or umbilical cord  Absence of stomach bubble on fetal sonography |
| Nervous system | Microcephaly  Hydrocephalus  Aplasia/Hypoplasia of corpus callosum  Abnormality of the septum pellucidum  Intracranial hemorrhage |
| Cardiovascular system | Abnormality of the pulmonary artery  Abnormality of the aorta  Tetralogy of fallot  Abnormality of the heart valves  Congenital malformation of the great arteries |
| Genitourinary system | Polycystic kidney  Renal cyst  Abnormal renal morphology  Ectopic/pelvic kidney  Abnormality of external genitalia  Abnormality of the urethra  Abnormality of the bladder  Abnormal cloaca  Abnormal renal number  Renal agenesis |
| Abdomen | Omphalocele  Abnormality of the abdominal organs  Abnormality of the liver  Pleural effusion |
| Digestive system | Echogenic bowel  Ectocolon |
| Multisystem | Involving>1 system |
