# Supplementary material for: Prenatal whole-exome sequencing for fetal structural anomalies: a retrospective analysis of 145 Chinese cases
Source: BMC Med Genomics. 2023 Oct 25;16:262. doi: 10.1186/s12920-023-01697-3 (PMC10601195; doi:10.1186/s12920-023-01697-3)
Supplement: Supplementary file 2 — Supplementary Material 2 [file 12920_2023_1697_MOESM2_ESM.docx]

**Table S2** Variants of uncertain significance

| **Case ID** | **Ultrasound findings** | **Gene** | **Variant** | **Inheritance/Zygosity** | **Disease Association(s)** |
| --- | --- | --- | --- | --- | --- |
| **2942** | Cleft lip and palate | *GLI2* | NM_005270.5  c.2629G>A  p.(G877R) | AD/het | Culler-Jones syndrome  OMIM:615849 |
| **2945** | Microcephaly, porencephaly | *L1CAM* | NM_001278116.2  c.1940-17G>A | XLR/hemi | CRASH syndrome  OMIM:303350 |
| **2909** | Microcheiria, flexion contracture | *MAGEL2* | NM_019066.5  c.444_503del  p.(S150_M169del20) | AD/het | Schaaf-Yang syndrome  OMIM:615547 |
| **2074** | Abnormality of external genitalia, cardiac abnormality, seroperitoneum | *MED12* | NM_005120.3  c.204+61C>T | XLR/hemi | Opitz-Kaveggia syndrome  OMIM:305450 |
| **5547** | Ventricular septal defect, microcephaly | *PTCH1* | NM_000264.5  c.892A>G  p.(N298D) | AD/het | Holoprosencephaly 7  OMIM:610828 |
| **6424** | Severe hydrocephalus, left finger deformity, cleft lip and palate | *COL11A1* | NM_001854.4  c.4495C>T  p.(P1499S)  c.4518+14A>G | AD/AR/het | Fibrochondrogenesis 1  OMIM:228520  Marshall syndrome  OMIM:154780 |
| 2971 | Agenesis of corpus callosum, polyhydramnios, microphthalmia | *FIG4* | NM_014845.5  c.658A>G  p.(I220V)  c.2327C>T  p.(S776F) | AR/het | Yunis-Varon syndrome  OMIM:216340 |
| 3073 | Syndactyly, missing finger deformity | *FAT4* | NM_024582.4  c.14429A>G  p.(H4810R)  c.14929G>A  p.(A4977T) | AR/het | Van Maldergem  syndrome 2  OMIM:615546 |
| 3042 | Edema, pleural effusion, embryo stasis | *NEB* | NM_001271208.2  c.12316G>A  p.(D4106N)  c.9015T>A  p.(N3005K) | AR/het | Nemaline myopathy 2, autosomal recessive  OMIM:256030 |
| 3053 | Polycystic kidney | *CEP290* | NM_025114.4  c.2638G>T  p.(A880S)  c.620A>G:  p.(Q207R) | AR/het | Joubert syndrome 5  OMIM:610188 |
| 6440 | Small mandible, bilateral choroid plexus cysts, scoliosis, ventricular septal defect, single umbilical artery | *KMT2C* | NM_170606.3  c.12163A>G  p.(I4055V) | AD/het | Kleefstra syndrome 2  OMIM:617768 |
| 8202 | Tetralogy of Fallot, esophageal atresia, small gastric alveoli, left ear redundancy | *TRAF7* | NM_032271.3  c.61A>G  p.(T21A) | AD/het | Cardiac, facial, and  digital anomalies with developmental delay  OMIM:618164 |
| 8219 | Cervical lymphoedema, abdominal wall defect, scoliosis, short umbilical cord | *MAP2K2* | NM_030662.3  c.450+6T>G | AD/het | Cardiofaciocutaneous syndrome 4  OMIM:615280 |

Abbreviations: AD, autosomal dominant; AR, autosomal recessive; XLR, X-linked recessive; Hemi, hemizygous; Het, heterozygous. Bolded IDs indicated the trio WES cases.
