# Supplementary figures and images for: Prenatal whole-exome sequencing for fetal structural anomalies: a retrospective analysis of 145 Chinese cases
Source: BMC Med Genomics. 2023 Oct 25;16:262. doi: 10.1186/s12920-023-01697-3 (PMC10601195; doi:10.1186/s12920-023-01697-3)

**Supplementary file 1** Sanger sequencing validation of the variants for the proband-only cases


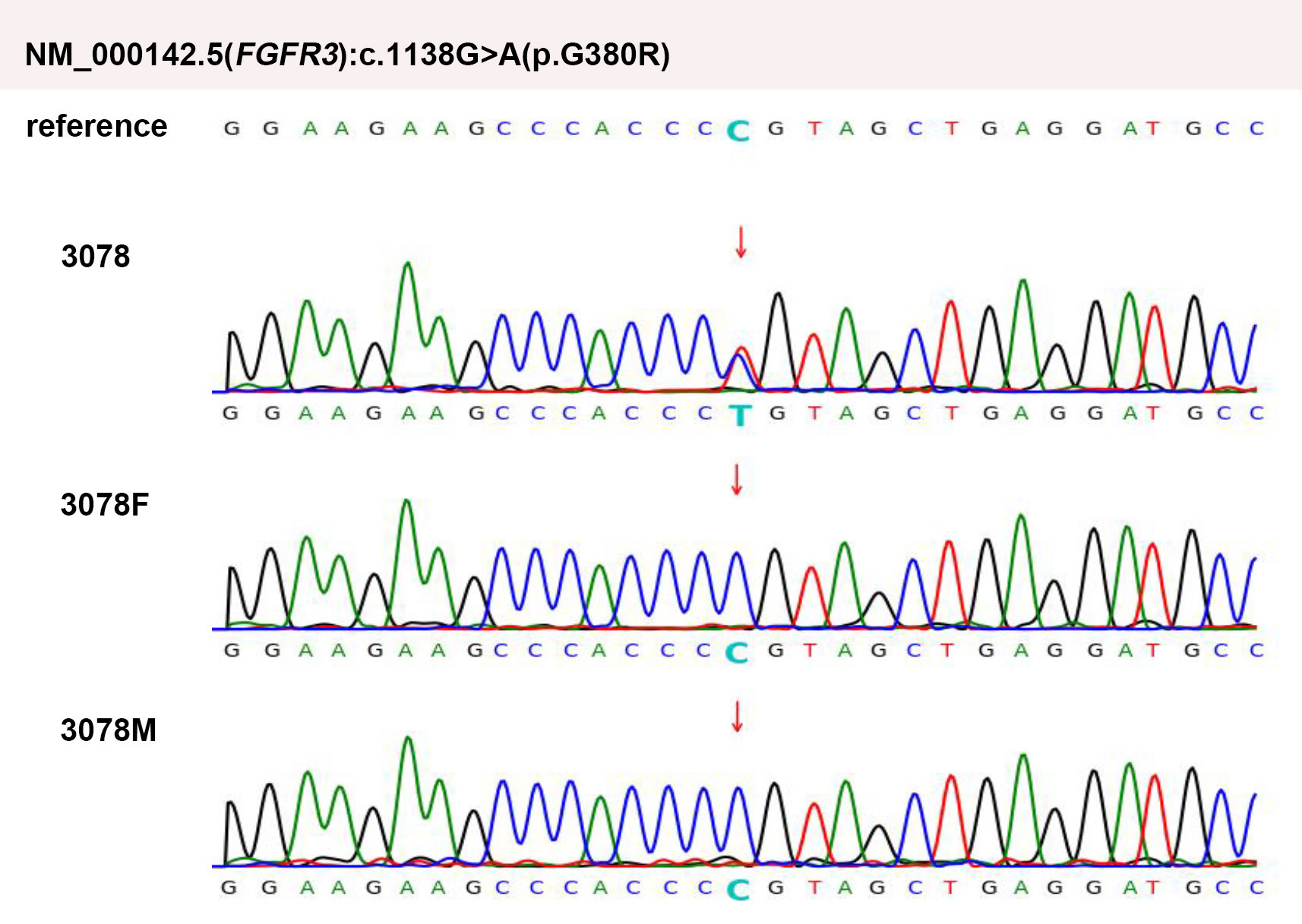


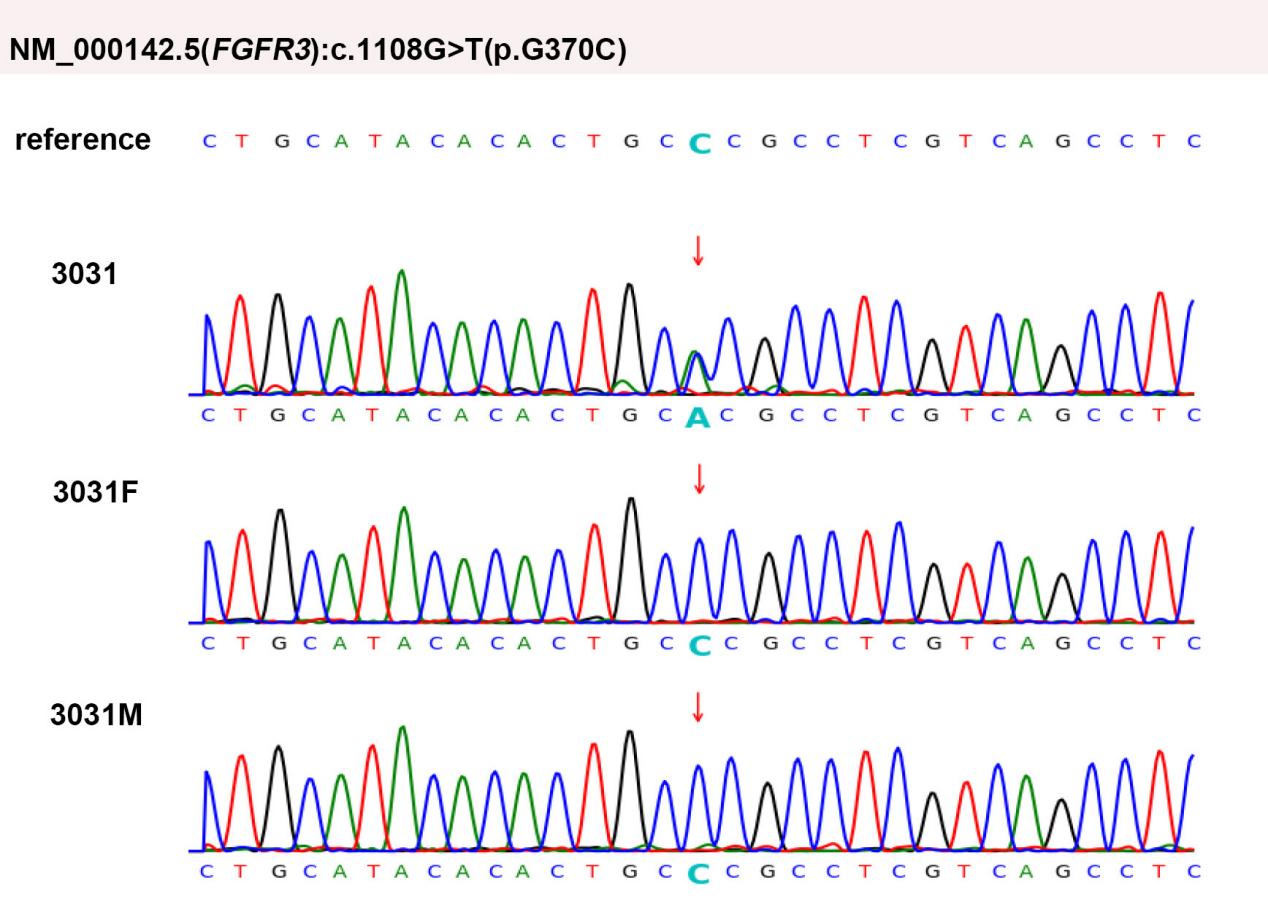


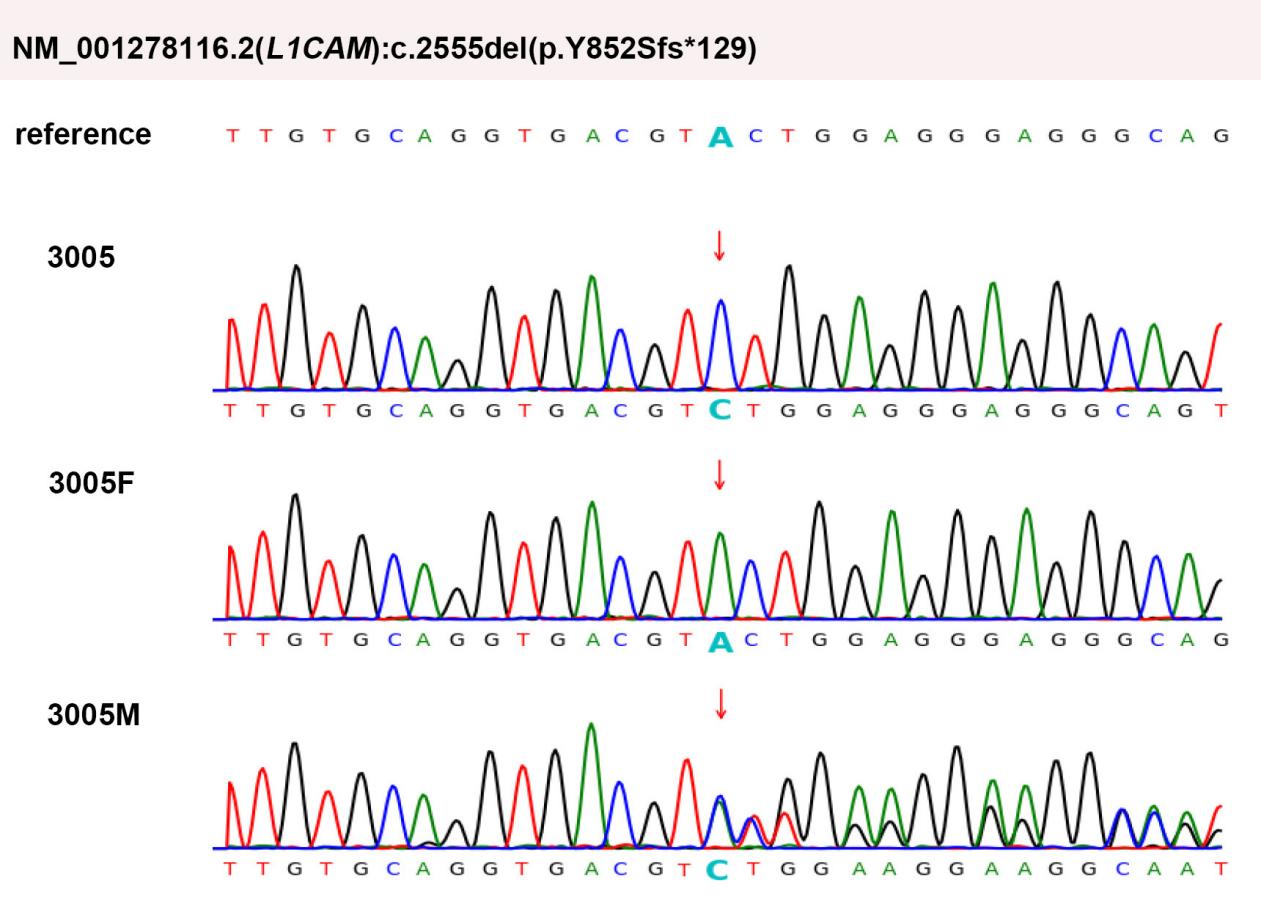


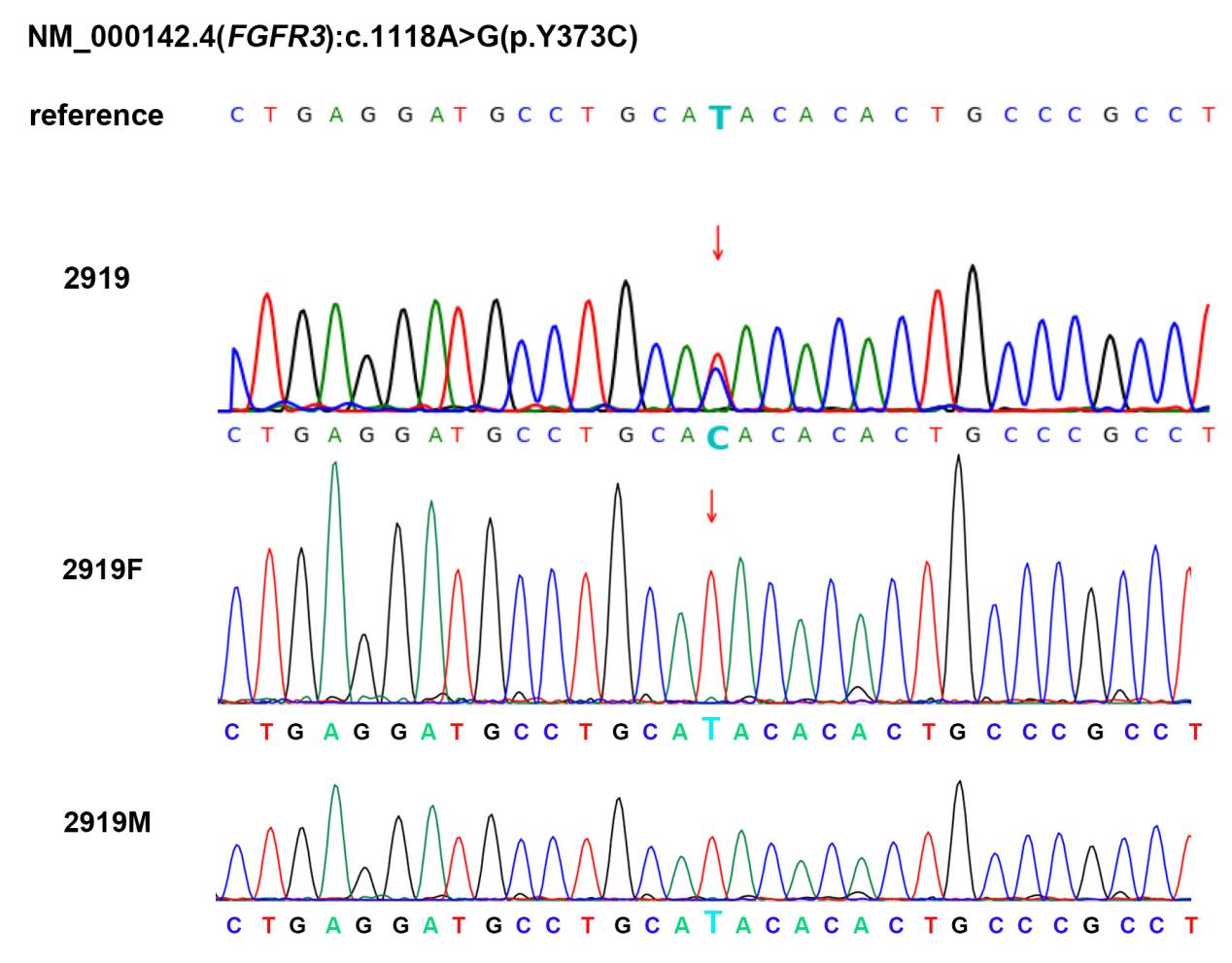


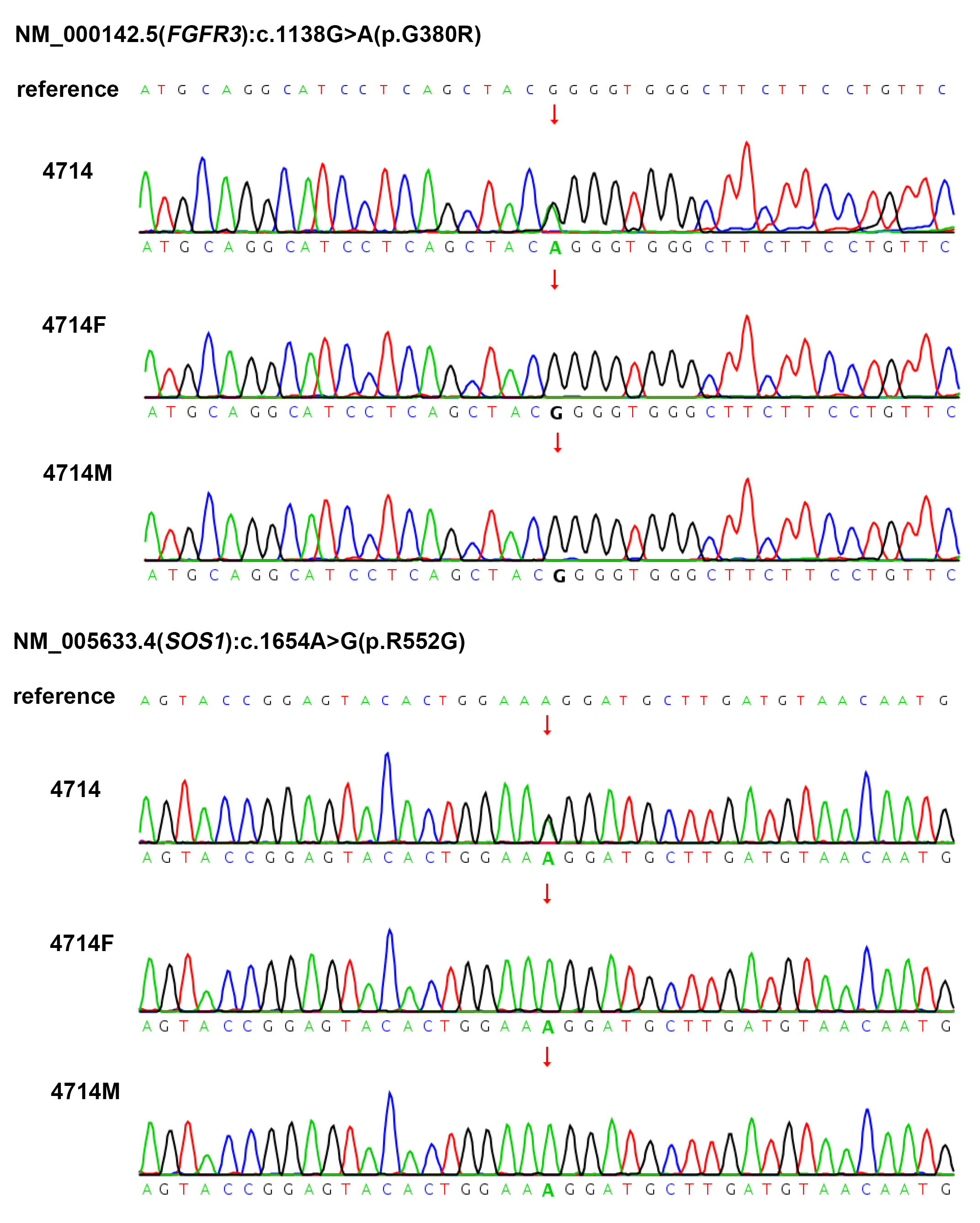


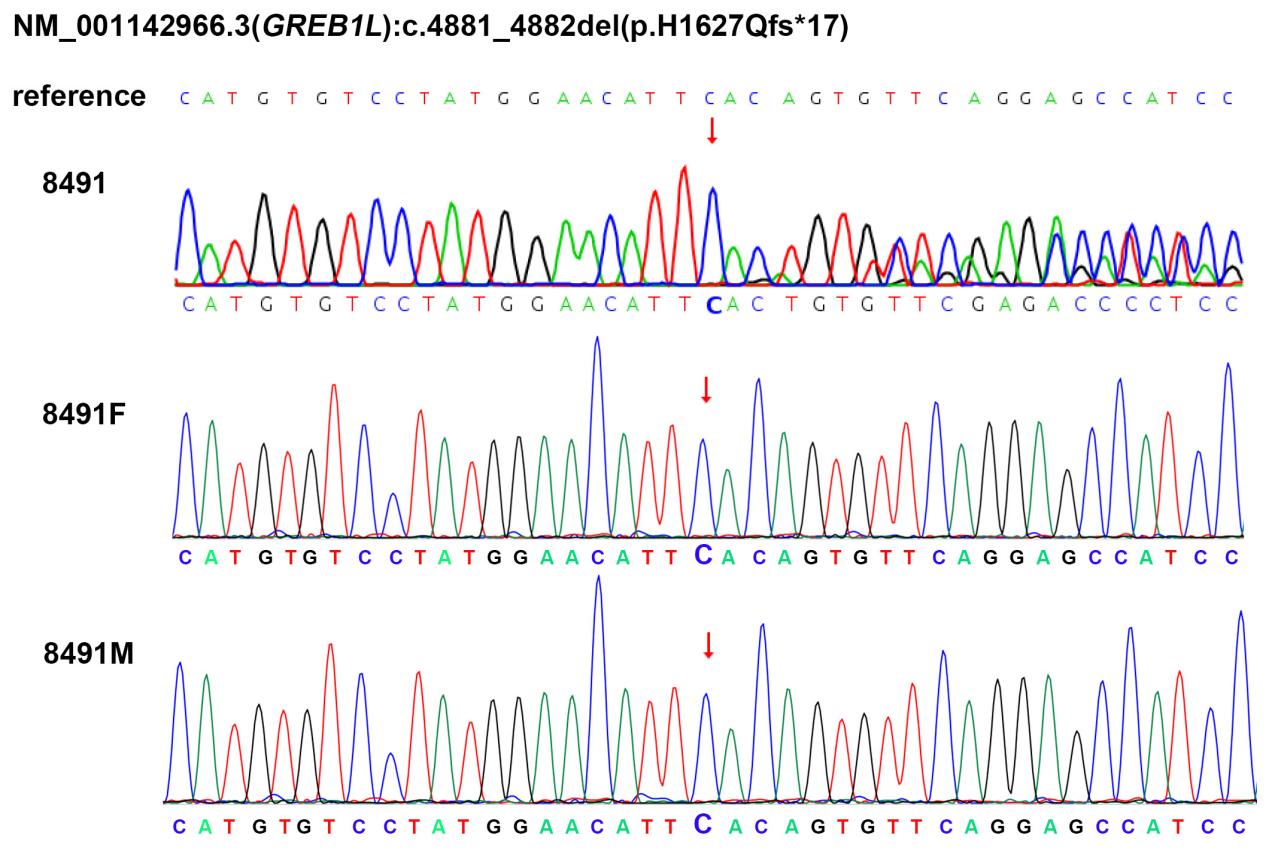


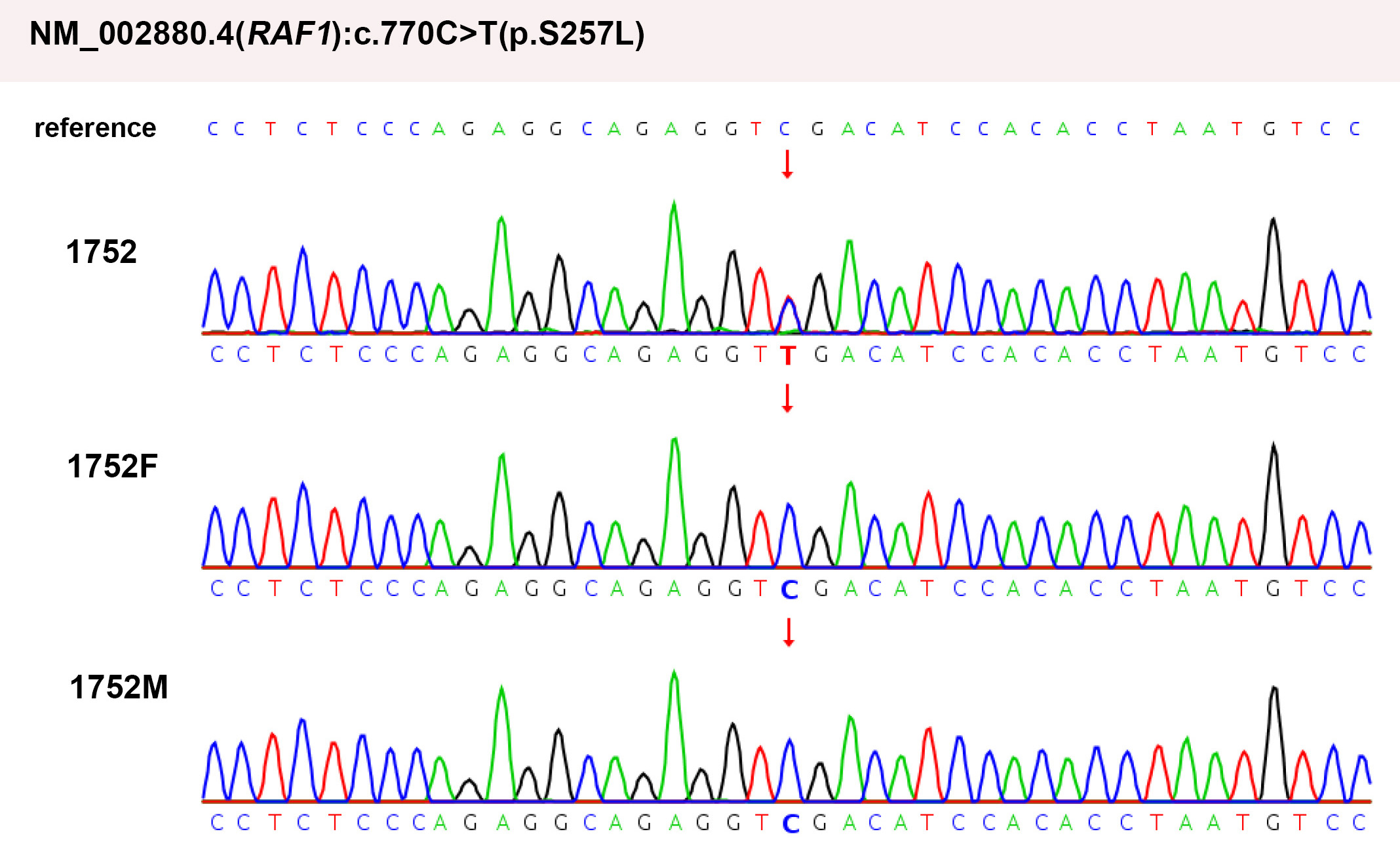


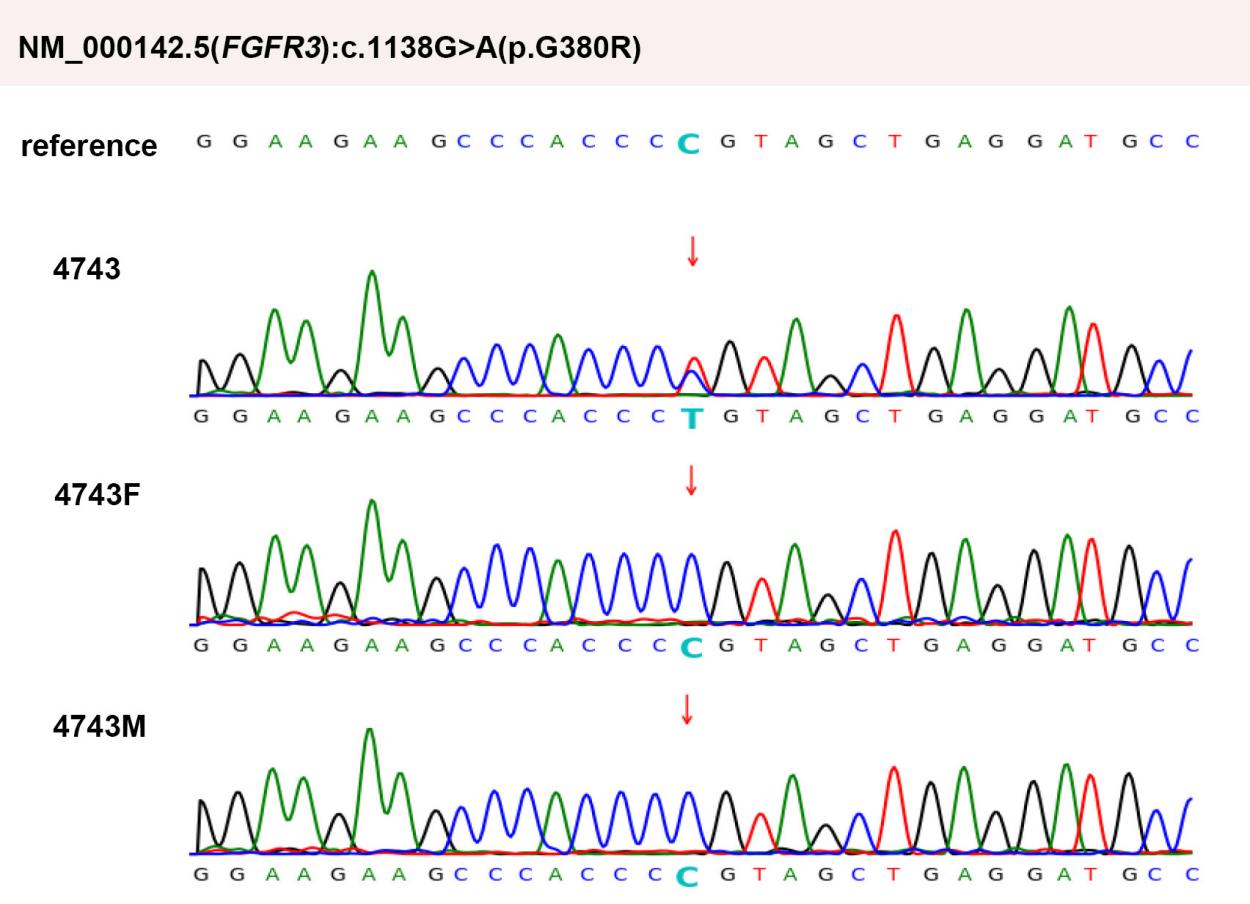

Supplement: Supplementary file 3 — Supplementary Material 3 [file 12920_2023_1697_MOESM3_ESM.docx]

**Supplementary file 2** Validating SMPD4 deletion using qPCR for the 2014 case
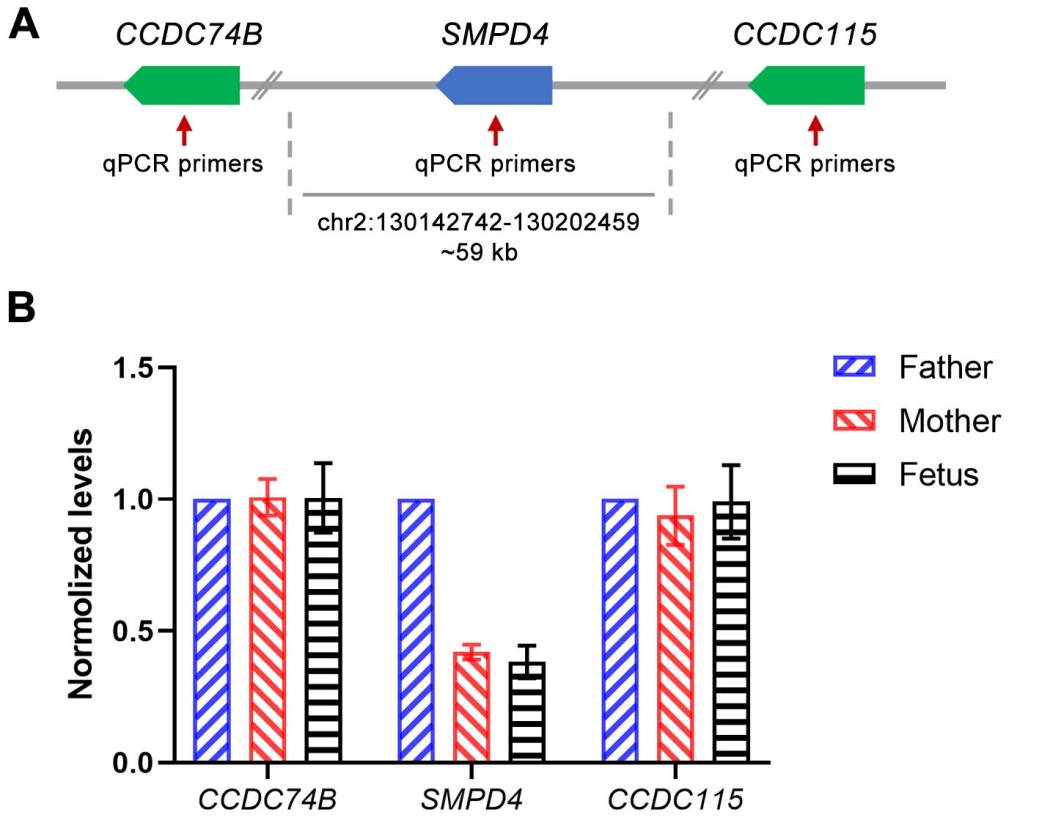

Supplement: Supplementary file 4 — Supplementary Material 4 [file 12920_2023_1697_MOESM4_ESM.docx]
